# Clinical outcomes of topography-guided femtosecond laser-assisted in situ keratomileusis after multifocal intraocular lens implantation

**DOI:** 10.1038/s41598-020-67726-8

**Published:** 2020-06-30

**Authors:** Eunhae Shin, Young-Sik Yoo, Sung-Ho Choi, Sun-Hyup Han, Dong Hui Lim, Gil-Joong Yoon, Tae-Young Chung

**Affiliations:** 1 Department of Ophthalmology, Sungkyunkwan University School of Medicine, Samsung Medical Center, Seoul, Republic of Korea; 2 Department of Ophthalmology, College of Medicine, Uijeongbu St. Mary’s Hospital, The Catholic University of Korea, Uijeongbu, Gyeonggi-do Republic of Korea; 3 BALGEUN-EYE21 Hospital, Gwangju, Republic of Korea; 4 Department of Medical Device Management and Research, Samsung Advanced Institute for Health Science and Technology, Sungkyunkwan University School of Medicine, Seoul, Republic of Korea

**Keywords:** Medical research, Eye diseases

## Abstract

This retrospective study is to evaluate refractive and visual outcomes of topography-guided femtosecond laser-assisted in situ keratomileusis (TGL) for correcting corneal high-order aberrations (HoA) after multifocal intraocular lens (mIOL) implantation. Twenty-eight eyes of 28 patients with both corrected distance visual acuity (CDVA) under 20/25 and subjective visual discomfort at 3 months after mIOL implantation were included in the study. TGL was performed to correct corneal HoA. Visual acuity, manifest refraction, and corneal HoA were measured 3 months after TGL. CDVA was improved in 22 (78.57%) of 28 eyes after TGL. Uncorrected distance visual acuity (0.12 ± 0.16 logMAR) and uncorrected near visual acuity (0.081 ± 0.16 logMAR) were better than those before TGL (*P* < 0.001). Residual refractive astigmatism showed no difference compared to that before TGL. Root mean square (RMS) of HoA (*P* = 0.012), spherical aberration (*P* = 0.013), and RMS of coma (*P* = 0.001) were reduced relative to those before TGL. Amount of improvement in CDVA was correlated with amount of reduced coma RMS (R = 0.524; *P* = 0.005) and spherical aberration (R = 0.443; *P* = 0.021). TGL showed to improve both refractive and visual outcomes in patients with mIOL implantation by correcting corneal HoA.

## Introduction

The number of cataract surgeries conducted involving multifocal intraocular lenses (mIOLs) has been increasing due to the resultant improvement of both distant and near visual outcomes. However, some patients experience unsatisfied visual acuity or dysphotopsia after mIOL implantation due to refractive errors such as residual ametropia or astigmatism and corneal high-order aberration (HoA)^1,2^. To meet patient’s expectations for clear vision without spectacles, several attempts have been made to improve refractive error after mIOL implantation. Surgical methods like laser-assisted in situ keratomileusis (LASIK) or photorefractive keratectomy (PRK) have been emphasized to successfully correct residual refractive error after mIOL surgery^3^.

Conventional LASIK and PRK are known to significantly increase corneal HoA including coma and spherical aberrations^4^. There have been attempts to minimize induction of HoA using wavefront-guided LASIK (WGL) or wavefront-optimized LASIK (WOL). Previous studies have reported that WGL induced less corneal HoA compared to WOL^5,6^ and conventional LASIK^7^. Nevertheless, WGL cannot completely eliminate residual HoA after mIOL implantation because changable pupil size and slight eye movements during the wavefront measurement can result in inconsistent preoperative corneal HoA measurements, which open the potential for error^8^.

To overcome such a predicament, topography-guided femtosecond-LASIK (TGL) was introduced recently to correct irregular astigmatism on the corneal surface by separately calculating corneal HoA^9^. Assuming internal aberrations do not affect postoperative visual outcomes, TGL is expected to be superior to WGL for correcting corneal HoA, which represents a relatively consistent values during preoperative evaluation and accounts for most of the total ocular aberrations of the eye. To our knowledge, the relationship between correction of residual corneal HoA and visual outcomes after TGL in patients with mIOL implantation has not been reported (source: PubMed; Keywords: topography, LASIK, HoA, multifocal). The present study evaluated refractive and visual outcomes of TGL for correcting corneal HoA after mIOL implantation.

## Methods

This retrospective study reviewed electronic medical records of patients who underwent TGL after mIOL implantation to correct corneal HoA and improve visual acuity. Cataract surgeries using mIOL (SN6AD1; Alcon, Fort Worth, TX, USA), an apodized diffractive aspheric mIOL with a + 3.0 diopter addition power^10^, were conducted from April 2015 to September 2017 (single surgeon). Procedures involving TGL after cataract surgery were conducted from November 2016 to May 2018.

The present study included patients whose corrected distance visual acuity (CDVA) was less than 20/25 for at least 3 months (mean: 196.34 days, standard deviation: 176.47 days) after mIOL implantation without specific findings including posterior capsular opacity or any pathologic findings on their maculas. Cases in which a large amount of corneal HoA was thought to be the main cause of subjective discomfort such as sustained monocular diplopia or glare and halos were included and underwent TGL. Patients who showed an improvement in dry eye disease or inflammation of the eye after proper medications over 3 months were excluded from the present study. Patients with special conditions contraindicating laser ablation were also excluded (e.g., low central corneal thickness, keratoconus, recurrent corneal erosion). The present study analyzed 28 eyes of 28 patients. The mean age of study subjects was 56.9 ± 10.0 (SD) years (Table 1). The present study was approved by the Institutional Review Board (IRB) of Samsung Medical Center (IRB no. 2019-09-065) and adhered to the tenets of the Declaration of Helsinki. Informed consent was exempted by IRB of Samsung Medical Center (IRB no. 2019-09-065).

**Table 1:** Summary of demographic and preoperative biometric data.

Number	28 eyes
Age (years)	56.9 ± 10.0
Sex (female, %)	5 (17.9%)
Laterality (right eye, %)	19 (53.6%)
UDVA (logMAR)	0.31 ± 0.13
CDVA (logMAR)	0.16 ± 0.092
Manifest refraction spherical equivalent (D)	− 0.29 ± 0.54
Refractive sphere	0.28 ± 0.66
Refractive astigmatism	− 1.13 ± 0.91
**Topography measurements (D)**	
Anterior corneal astigmatism	− 1.00 ± 0.61
Corneal high-order aberration (RMS)	0.75 ± 0.36

Corneal aberrations were measured across a 6.0-mm diameter scan site.

*UDVA* uncorrected distance visual acuity, *CDVA* corrected distance visual acuity, *D* Diopter, *RMS* root mean square.

### Patient examinations

Preoperative (pre-TGL) visual acuity was measured including corrected distance visual acuity (CDVA), corrected near visual acuity (CNVA), uncorrected distance visual acuity (UDVA), and uncorrected near visual acuity (UNVA). To plan the amount of laser ablation to the cornea, manifest refraction and corneal topography were evaluated. Corneal HoA, and pupil size were obtained from topographic data measured by Topolyzer VARIO (WaveLight; Alcon, Fort Worth, TX, USA) before TGL. Preoperative corneal thickness was measured using ultrasonic pachymeter (Pocket-II, Quantel medical, France). Postoperative (post-TGL) measurements performed were visual acuity (UDVA, CDVA, UNVA, and CNVA), manifest refraction, and topographic data at 3 months after TGL.

### Surgical technique

All TGLs were performed by one surgeon at least 3 months after mIOL implantation. Topographic data on the date of the surgery were transmitted to the excimer laser software program (Wavelight EX500; Alcon, Fort Worth, TX, USA) via a wireless network connection. Based on the calculated mean value in topo-guided surgery mode, at least four repeatable topographic data points were used. These points were within the central 6.5-mm zone when the gap between the K1 and K2 values was less than 0.25 D and the axis of the steep meridian was within two degrees from the average. Both the amount and axis of astigmatism correction for laser ablation was determined based on manifest refraction measurements.

First, a corneal flap (thickness: 105 μm, radius: 9 mm, hinge at 12 o/c) was created with femtosecond laser (Wavelight FS200; Alcon, Fort Worth, TX, USA). The optic zone was 6.0 mm in corneal ablation. After ablation, the stromal bed was irrigated with balanced salt solution (BSS Sterile Irrigating Solution; Alcon, Fort Worth, TX, USA), and the flap was returned to the original position. A therapeutic bandage contact lens (ACUVE Oasys lens, Johnson and Johnson Vision Care, Inc. Jacksonville, FL, USA) was applied and removed on the first postoperative day. Flumetholone (0.1% Flumetholone; Santen Pharmaceutical Co., Ltd., Osaka, Japan) and Vigamox (moxifloxacin; Alcon, Fort Worth, TX. USA) were used postoperatively four times a day for 2 weeks. Preservative-free, artificial eyedrops containing hyaluronic acid were used every 2 h for 3 months after TGL. Reoperation was conducted if visual acuity had not improved at 3 months postoperatively.

## Results

Pre-TGL UDVA and CDVA were 0.31 ± 0.13 and 0.16 ± 0.092 logMAR, respectively. Refractive astigmatism and anterior corneal astigmatism before TGL were − 1.13 ± 0.91 and − 1.00 ± 0.61 D, respectively. Preoperative root mean square (RMS) of total corneal HoA was 0.74 ± 0.37.

UDVA, CDVA, UNVA, and CNVA after TGL were significantly improved relative to those before TGL (*P* < 0.05) (Table 2). Meanwhile, the refractive cylinder was − 1.13 ± 0.91 D preoperatively and − 0.64 ± 0.39 D (*P* = 0.128) postoperatively. Preoperative and postoperative anterior corneal astigmatism readings from topography measurements were − 1.00 ± 0.61 D and − 0.67 ± 0.43 D (*P* = 0.039), respectively.

**Table 2:** Visual and refractive outcomes before and at 3 months after topography guided LASIK.

	Preoperative	Postoperative	*P *value^a^
UDVA (logMAR)	0.31 ± 0.13	0.12 ± 0.16	< 0.001^b^
CDVA (logMAR)	0.16 ± 0.092	0.063 ± 0.11	0.001^b^
UNVA (logMAR)	0.22 ± 0.20	0.081 ± 0.16	< 0.001^b^
CNVA (logMAR)	0.17 ± 0.18	0.056 ± 0.15	0.007^b^
MRSE (D)	− 0.29 ± 0.54	− 0.29 ± 0.41	0.131
Refractive sphere	0.28 ± 0.66	0.036 ± 0.46	0.016^b^
Refractive astigmatism	− 1.13 ± 0.91	− 0.64 ± 0.39	0.128
**Topography measurements (D)**			
Anterior corneal astigmatism	− 1.00 ± 0.61	− 0.67 ± 0.43	0.039^b^

*UDVA* uncorrected distance visual acuity, *CDVA* corrected distance visual acuity, *UNVA* uncorrected near visual acuity, *CNVA* corrected near visual acuity, *MRSE* manifest refraction spherical equivalent, *D* Diopter.

^a^Wilcoxon signed ranks test.

^b^Statistically significant.

**Figure 1:**
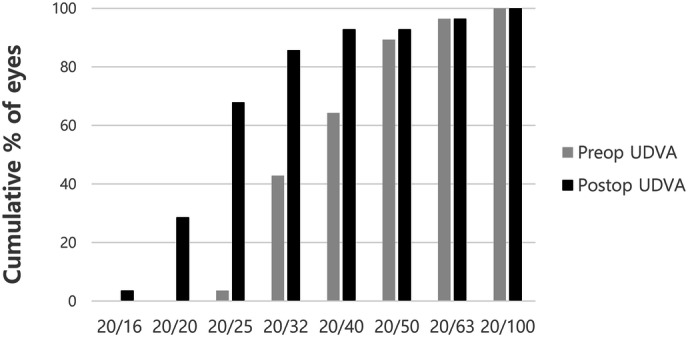
Cumulative unilateral Snellen uncorrected distance visual acuity before and at 3 months after topography guided LASIK. *UDVA* uncorrected distance visual acuity.

**Figure 2:**
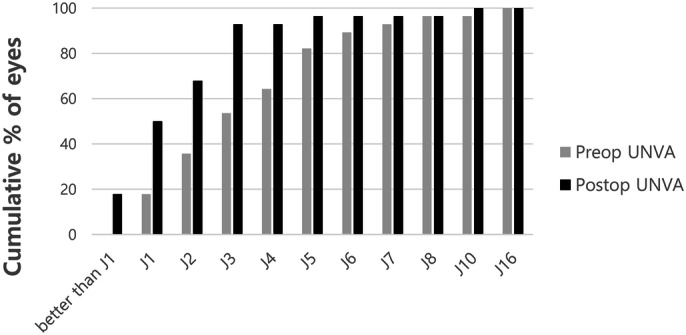
Cumulative unilateral Snellen uncorrected near visual acuity (UNVA) before and at 3 months after topography guided LASIK. *UNVA* uncorrected near visual acuity.

**Figure 3:**
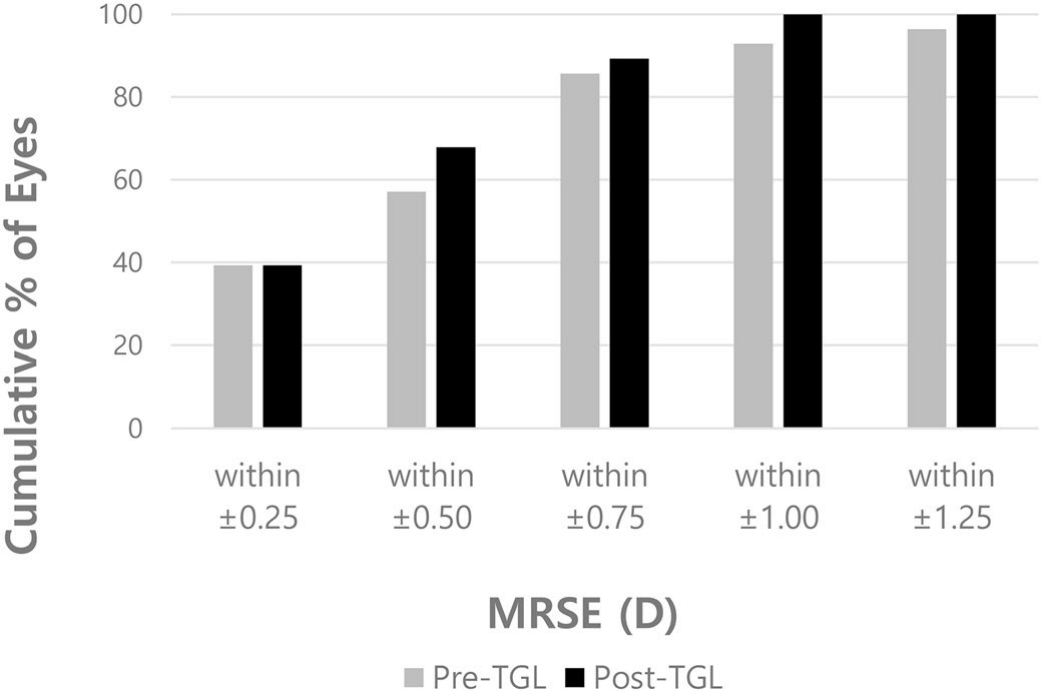
Cumulative manifest refractive spherical equivalent before and at 3 months after topography guided LASIK (TGL). *MRSE* manifest refractive spherical equivalent, *D* Diopter.

**Figure 4:**
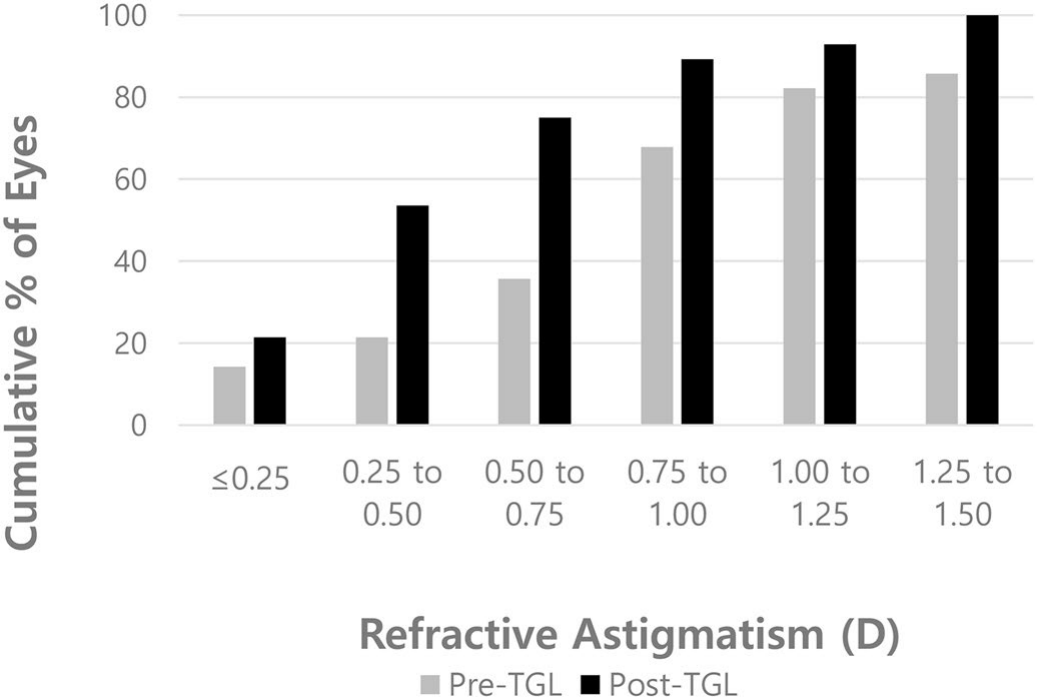
Cumulative refractive astigmatism outcomes before and at 3 months after topography guided Laser in situ keratomileusis. *TGL* topography guided femtosecond laser-assisted in situ keratomileusis, *D* Diopter.

**Figure 5:**
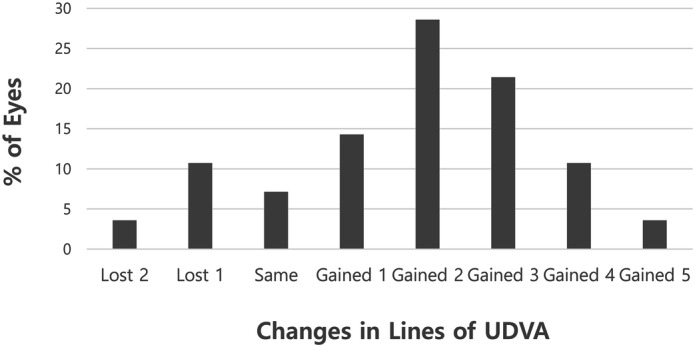
Difference between uncorrected distance visual acuity before and 3 months after topography guided Laser in situ keratomileusis. *UDVA* uncorrected distance visual acuity.

Cumulative postoperative unilateral UDVA was 20/20, 20/25, 20/32, and 20/40 in 28.6%, 67.9%, 85.7%, and 92.9% of eyes, respectively (Fig. 1). Cumulative postoperative unilateral UNVA was J1, J3, and J5 in 50.0%, 92.9%, and 96.4% of eyes, respectively (Fig. 2). The percentage of eyes with manifest refractive spherical equivalent within ± 1.0 D was 92.9% preoperatively and 100% after TGL (Fig. 3). The percentage of eyes with refractive astigmatism within 1.0 D was increased from 67.9 to 89.3% after TGL (Fig. 4). Twenty-two of 28 eyes (78.6%) showed improved CDVA after TGL, two remained the same, and four eyes worsened after TGL (Fig. 5).

**Figure 6:**
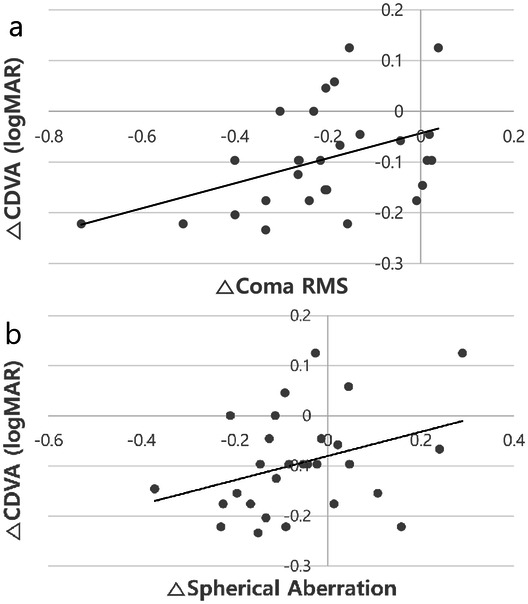
Correlation between changes of coma root mean square (**a**) or spherical aberration (**b**) and change of corrected distance visual acuity. *CDVA* corrected distance visual acuity, *RMS* root mean square.

Corneal aberrations were measured at 3 months after TGL. Preoperative high-order aberration root mean square (RMSh) and postoperative RMSh were 0.74 ± 0.37 and 0.54 ± 0.30, respectively (*P* = 0.012) (Table 3). Spherical aberration (0.16 ± 0.15 preoperatively and 0.072 ± 0.19 postoperatively; *P* = 0.013) and coma RMS (0.46 ± 0.26 preoperatively and 0.24 ± 0.16 postoperatively; *P* = 0.001) were also decreased after TGL. Although trefoil RMS (*P* = 0.502) showed no difference after TGL, oblique trefoil **(**${{\varvec{Z}}}_{3}^{-3}$**)** (− 0.10 ± 0.29 preoperatively and 0.024 ± 0.28 postoperatively; *P* = 0.032) decreased after TGL. Difference between postop and preop CDVA logMAR had positive correlation with both changes of coma RMS (R = 0.524; *P* = 0.005) (Fig. 6a) and spherical aberration RMS (R = 0.443; *P* = 0.021) (Fig. 6b).

**Table 3:** Corneal high-order aberrations before and at 3 months after topography guided LASIK.

	Preoperative	Postoperative	*P *value^a^
**Corneal aberrations**			
Total high-order aberrations (RMSh)	0.74 ± 0.37	0.54 ± 0.30	0.012^b^
Spherical aberration (${{\varvec{Z}}}_{4}^{0}$)	0.16 ± 0.15	0.072 ± 0.19	0.013^b^
Coma (RMS)	0.46 ± 0.26	0.24 ± 0.16	0.001^b^
Vertical coma (${{\varvec{Z}}}_{ 3}^{-1}$)	− 0.0095 ± 0.38	0.050 ± 0.22	0.781
Horizontal coma (${{\varvec{Z}}}_{3}^{1}$)	0.00028 ± 0.36	− 0.0010 ± 0.17	0.502
Trefoil (RMS)	0.36 ± 0.28	0.28 ± 0.25	0.108
Oblique trefoil (${{\varvec{Z}}}_{ 3}^{-3}$)	− 0.10 ± 0.29	0.024 ± 0.28	0.032^b^
Horizontal trefoil (${{\varvec{Z}}}_{3}^{3}$)	− 0.039 ± 0.34	0.072 ± 0.19	0.001^b^

Corneal aberrations were measured across a 6.0-mm diameter scan site.

*RMSh* high-order aberration root mean square, *RMS* root mean square.

^a^Wilcoxon signed ranks test.

^b^Statistically significant.

Six of 28 eyes underwent reoperation (TGL) due to unsatisfactory visual outcomes. Three eyes showed no improvement in either CDVA or CNVA after the first TGL operation. Although both CDVA and CNVA of the other three eyes improved, they remained worse than 20/25 (Snellen) and J2 (Jaeger) after the first TGL, respectively. As a result of the second TGL, all eyes showed improvement in both CDVA and CNVA. Both CDVA and CNVA of four eyes were better than 20/25 and J2, respectively, while the other two eyes showed 20/30 and J4. Re-TGL reduced both refractive sphere and astigmatism from to − 0.50 ± 0.60 D and − 1.04 ± 0.30 D to − 0.041 ± 0.39 D and − 0.54 ± 0.39 D, respectively.

## Discussion

In our institution, we found out that some of the patients who underwent cataract surgery with mIOL had not satisfied with their visual quality. Those patients showed some degree of refractive astigmatism and corneal aberrations. The surgeon tried TGL to correct residual refractive error and corneal aberration for visual acuity improvement. Authors conducted this retrospective study to find out whether TGL had effectively corrected both factors. In the present study, TGL after mIOL implantation showed significant improvement in both refractive and visual outcomes. TGL was effective in reducing corneal HoA such as RMSh, spherical aberration, coma RMS, and oblique trefoil and in improving visual acuity for both far and near distance.

MIOLs inherently demonstrate worse optical qualities than monofocal IOLs. These devices split the available light into far-vision and near-vision, increasing corneal HoA and, therefore, decreasing quality of vision. Major causes of poor visual quality include photopic side effects such as nighttime visual acuity loss, glare, halo and low contrast sensitivity^11,12^. Such side effects are more prominent in relation to mIOLs with corneal shape irregularity and astigmatism greater than 1 D^13^. A study reported higher objective scatter index (OSI) values of mIOL (SN6AD1; Acrysof IQ ReSTOR, Alcon Inc., Fort Worth, TX, USA) relative to monofocal IOL (SN60WF; Acrysof IQ, Alcon Inc., Fort Worth, TX, USA)^14^. Such increase in corneal HoA after mIOL implantation is one of the main predicaments that needs to be overcome.

Conventional LASIK or PRK has been used to correct postoperative refractive error after mIOL implantation^15,16^, but increased HoA after refractive surgery^4,17^ may facilitate subjective visual discomfort such as low visual acuity or dysphotopsia. To overcome such difficulties, wavefront-guided ablation (WGA) was introduced to reduce HoA in patients with a large amount of pre-existing HoA^5,6^. Theoretically, ablation of the anterior corneal surface with WGA may compensate for internal aberrations of the eye. However, attempts to correct high HoA yielded frustrating results because preoperative measurements of ocular HoA were unreliable, and factors such as epithelial hyperplasia, stromal remodeling, and tear film changes limited precise ablation. Moreover, WGA can change the path of light through the eye, altering the contribution of ocular HoA and, as a result, was found to increase RMSh^7,9,18^. Jendritza et al.^8^ did not recommend WGL after mIOL implantation due to unreliable measurement of both refraction and HoA.

The outcome of this study suggests the TGL can be an ideal method by which to correct ocular aberrations in patients with mIOL. Most of ocular HoA are derived from the cornea, and internal aberrations associated with positive function of mIOL are not altered by TGL. Reinstein et al.^19^ suggested that TGL is an effective tool of reoperation for patients with complaints of night vision disturbances after WGL by effectively enlarging and recentering the optical zone and reducing HoA. In another study comparing TGL and conventional LASIK, the RMS values for total coma and spherical aberration were significantly higher in eyes treated with conventional LASIK at 3 months of follow-up^20^. In the present study, six eyes (21.4%) with low visual acuity after the first TGL showed significant improvement after the second TGL. The large number of corneal HoAs that develops irregularities in corneal shape is an indication for TGL to improve visual quality.

In the present study, mean refractive sphere were reduced significantly (Table 2) and spherical aberration were reduced (negative shift) significantly (Table 3) compared to those before TGL. There is a possibility that the improvement of visual outcome was affected by the correction of refractive spherical error before TGL in our results. Although previous studies with various LASIK technique effectively corrected refractive error after mIOL implantation^15,16^, HoA were increased. Therefore, our results were meaningful that TGL improved both refractive spherical error and corneal HoA.

The present study assessed the effect of TGL in patients with a large amount of corneal HoA after mIOL implantation. If there is a significant amount of HoA without apparent ocular conditions that could affect visual acuity, TGL could be an effective treatment option for improving visual outcomes.
